# Chelation‐Driven Dual‐Interface Regulation for Long‐Life Aqueous Zn–I_2_ Batteries

**DOI:** 10.1002/advs.77565

**Published:** 2026-09-08

**Authors:** Dong Wook Kim, Seung Hwa Park, Tae Rim Kim, Gun Jang, Jun Su Kim, Myung‐Jun Kwak, Seokhwan Jin, Seongbin Ga, Chihyun Hwang, Ho Seok Park, Youngkwon Kim

**Affiliations:** ^1^ Advanced Batteries Research Center Korea Electronics Technology Institute Seongnam Gyeonggi‐do Republic of Korea; ^2^ School of Chemical Engineering Sungkyunkwan University Suwon Gyeonggi‐do Republic of Korea; ^3^ Department of Chemical Engineering College of Engineering Kyung Hee University Yongin Republic of Korea; ^4^ School of Chemical Engineering University of Ulsan Ulsan Republic of Korea; ^5^ School of Chemical Biological & Battery Engineering Gachon University Seongnam‐si Gyeonggi‐do Republic of Korea; ^6^ SKKU Institute of Energy Science and Technology (SIEST) Sungkyunkwan University (SKKU) Suwon Republic of Korea

**Keywords:** aqueous Zn–I_2_ battery, chelation chemistry, dual‐interface regulation, electrical double layer, polyiodide confinement

## Abstract

The growing demand for energy storage systems (ESS) driven by the expansion of renewable energy calls for aqueous Zn–I_2_ batteries that offer both safety and cost competitiveness. However, the hydrogen evolution reaction (HER) and Zn anode corrosion, combined with iodine dissolution and polyiodide shuttling at the I_2_ cathode, form a degradation loop through interelectrode interactions, that impairs battery lifespan and efficiency. This study proposes a dual‐interface regulation strategy by introducing the multidentate chelator TPEN as an electrolyte additive. This strategy promotes the formation of Zn^2+^–TPEN coordination species (TCC) in the electrolyte, which reconfigures the electric double layer of Zn anode into an interfacial environment with reduced water accessibility via interfacial adsorption, suppressing HER and corrosion, and stabilizing local pH fluctuations while promoting uniform nucleation and deposition. Simultaneously, at the I_2_ cathode, it traps polyiodide to reduce free polyiodide concentration and directs the reaction pathway toward the interface, thereby decreasing shuttling and self‐discharge while improving I_2_ deposition uniformity. As a result, the Zn||Zn symmetric cell operated stably for over 2000 h and achieved a Coulombic efficiency of over 96.2% and a capacity retention of 92.5% after 200 cycles under high‐loading I_2_ (5.5 mAh cm^−2^) and 0.18 C conditions.

## Graphical abstract

Zn^2+^–TPEN coordination generates TPEN‐derived coordination species (TCC) that contribute to the regulation of both electrode interfaces. At the Zn anode, it creates an interfacial environment with reduced water accessibility that suppresses HER and corrosion while promoting uniform deposition; at the I_2_ cathode, it captures polyiodide to inhibit shuttling and self‐discharge.

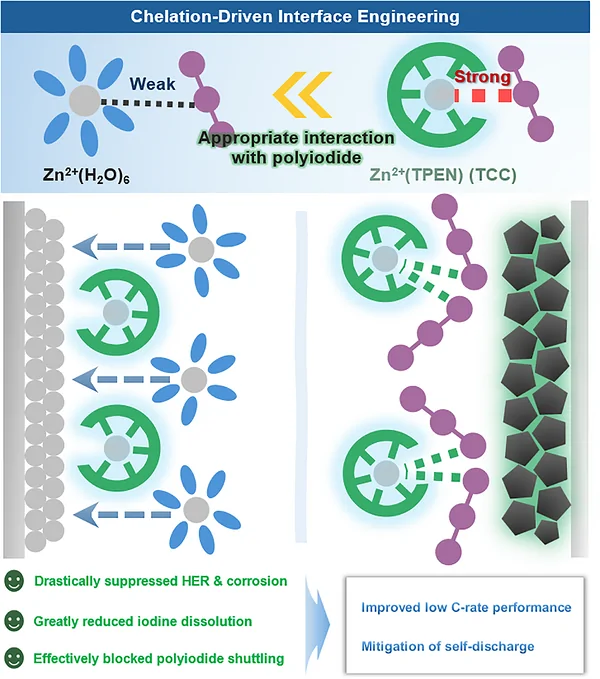

## 1 Introduction

As demand grows for long‐duration energy storage systems to buffer the intermittency and output variability of renewable energy generation, battery systems capable of high energy retention and long‐term durability under low C‐rate conditions—repeated charging and discharging over several hours or more—are required. In such applications, stability at current densities lower than instantaneous output, suppression of self‐discharge, and high active material utilization serve as key performance indicators. Aqueous zinc metal batteries are gaining attention as suitable candidates for large‐scale ESS due to their high safety based on non‐flammable electrolytes and cost competitiveness [1, 2, 3, 4, 5, 6, 7, 8]. Among them, aqueous Zn–I_2_ batteries stand out as promising options due to their relatively high operating voltage (≈ 1.3 V), high theoretical specific capacity (≈ 211 mAh g^−1^), and excellent safety features [9, 10, 11, 12]. However, under high iodine loading and low‐rate operating conditions, chemical side reactions occurring at both the anode and cathode accumulate over time, revealing a structural limitation that simultaneously impairs both lifespan and reversibility.

Under high‐loading conditions, the I_2_ concentration within the electrode and the polyiodide (I_3_^−^, I_5_^−^, etc.) concentration within the pore space increase, leading to a larger concentration gradient. During low C‐rate operation, polyiodides have sufficient time to pass through the separator and reach the Zn anode. The shuttling that occurs during this process goes beyond simple capacity loss; it induces chemical oxidation and irreversible depletion of the Zn anode, directly reducing Zn utilization. Simultaneously, at the Zn metal anode, the hydrogen evolution reaction (HER) and corrosion proceed over extended periods, causing localized pH increases and the accumulation of byproducts (e.g., ZHS) [13, 14, 15, 16]. These deposits create current distribution inhomogeneities, distort nucleation and growth behavior in subsequent cycles, and induce particularly critical inhomogeneities under high‐loading conditions. Consequently, under high‐loading, low C‐rate conditions, a mutually reinforcing degradation loop forms between the long‐range diffusion of polyiodides and the chemical depletion of Zn. This makes it difficult to achieve the high Coulombic efficiency and Zn utilization rate required for long‐duration ESS [17, 18, 19, 20, 21, 22, 23, 24].

To address these issues, various strategies for simultaneously controlling the anode and cathode have been reported. Notably, the Janus separator design, which incorporates different functional materials on each side of the separator, aims to independently stabilize each interface by trapping polyiodides on one side and regulating Zn^2+^ flux on the other [25, 26, 27]. While this approach demonstrated meaningful improvements in reducing shuttling and homogenizing Zn plating, it fundamentally relies on strategies that block migration pathways or physically mitigate interfacial phenomena. Under high‐loading, low C‐rate conditions, it fails to reduce the concentration of polyiodides continuously generated within the bulk electrolyte. Consequently, structural limitations emerge during long‐term operation, including saturation of the capturing layer, increased transport resistance, and sensitivity to coating uniformity and defects. Meanwhile, attempts to simultaneously regulate both anodes and cathodes via single electrolyte additives have been reported [28, 29, 30, 31, 32, 33, 34]. However, most approaches rely on charge‐based interactions or selective adsorption, partially mitigating specific interfacial reactions but remaining limited in scope. Particularly under high‐loading conditions, excessive polyiodide stabilization can hinder reversibility or cause sediment accumulation. Limitations persist in simultaneously suppressing HER and corrosion at the Zn interface while reducing shuttle driving force [35].

This study proposes a chelation chemistry‐based integrated interface control strategy utilizing the multidentate chelating molecule N,N,N′,N′‐tetrakis(2‐pyridylmethyl)ethylenediamine (TPEN) to overcome these structural bottlenecks [36, 37, 38, 39]. TPEN fundamentally modulates the nucleation and growth behavior, and HER reactivity at the cathode by forming strong coordination bonds with Zn^2+^ in the electrolyte, thereby reconfiguring the electrical double layer environment and ion transfer of Zn^2+^. Simultaneously, the formed TPEN–Zn^2+^ coordination species (TCC) readjusts the I_2_/I_x_^−^ equilibrium through electron donor–acceptor interactions with polyiodide, reducing the effective concentration and residence time of free polyiodide under high‐loading conditions. This strategy simultaneously redesigns the thermodynamic driving force and reaction pathways of the Zn^2+^‐I_x_^−^‐H_2_O chemical network, unlike approaches that rely solely on interfacial blocking or adsorption. Consequently, the single‐electrolyte design forms a molecular bridge connecting the cathode and anode, thereby weakening the dominant chemical degradation loop under high‐loading, low C‐rate conditions. This chelation chemistry‐based design differs from existing physical separation strategies using Janus separators or selective interfacial adsorption strategies, presenting a new electrolyte design paradigm to simultaneously achieve the high active material utilization and long‐term lifespan performance required for long‐cycle ESS.

## 2 Results and Discussion

### 2.1 Formation and Interfacial Adsorption of TPEN Chelating Complex

**FIGURE 1:**
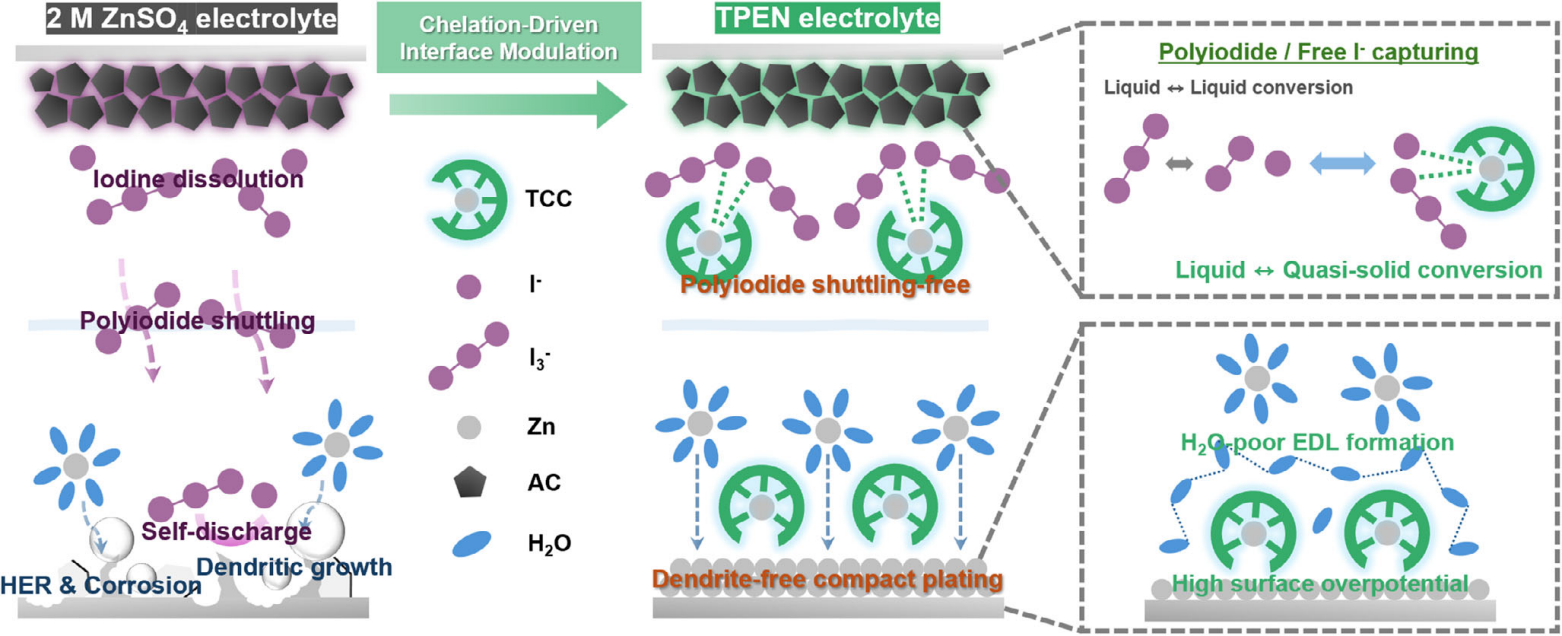
Schematic of interface‐stabilizing mechanism of TCC on both Zn anode and I_2_ cathode.

The core of the TPEN‐based interface design proposed in this study is that the TCC created within the electrolyte optimally influences the cathode and anode of Zn–I_2_ batteries. This enhances the interfacial stability of both electrodes simultaneously. Figure 1 outlines the conceptual framework of the dual‐interface control carried out by the TCC.

At the Zn metal anode, TCC selectively occupies the interface, restructuring the solvent and ion distribution within the electric double layer (EDL) and creating an interfacial environment with reduced water accessibility. This weakens the driving forces for hydrogen evolution and corrosion reactions while readjusting the interfacial potential gradient to facilitate a more uniform progression of initial nucleation processes. The protonatable donor sites of the TPEN buffer induce local pH fluctuations during Zn plating/stripping, suppressing the formation of basic byproducts (ZHS) and resulting in a homogeneous and dense Zn electrode morphology, ensuring high reversibility. At the I_2_ cathode, TCC reduces the effective concentration of freely diffusible shuttle species in the solution by selectively binding with polyiodide species, thereby alleviating the root causes of shuttling and self‐discharge. The TCC–polyiodide complex structure formed at the interface rearranges the reaction pathway, promoting the I_2_/I^−^ reaction near the electrode surface, which reduces concentration polarization and promotes spatially uniform I_2_ precipitation. This interface‐based reaction localization enhances the reversibility of I_2_ deposition/dissolution during charge and discharge cycles.

Consequently, while TCC performs distinct roles at the anode and cathode, it also functions as a molecular mediator that restores charge transfer equilibrium and suppresses crosstalk degradation between electrodes at the overall cell level. This strategy shows the potential of novel chelation‐driven molecular interface engineering to stabilize the anode–cathode interface with a single additive.

**FIGURE 2:**
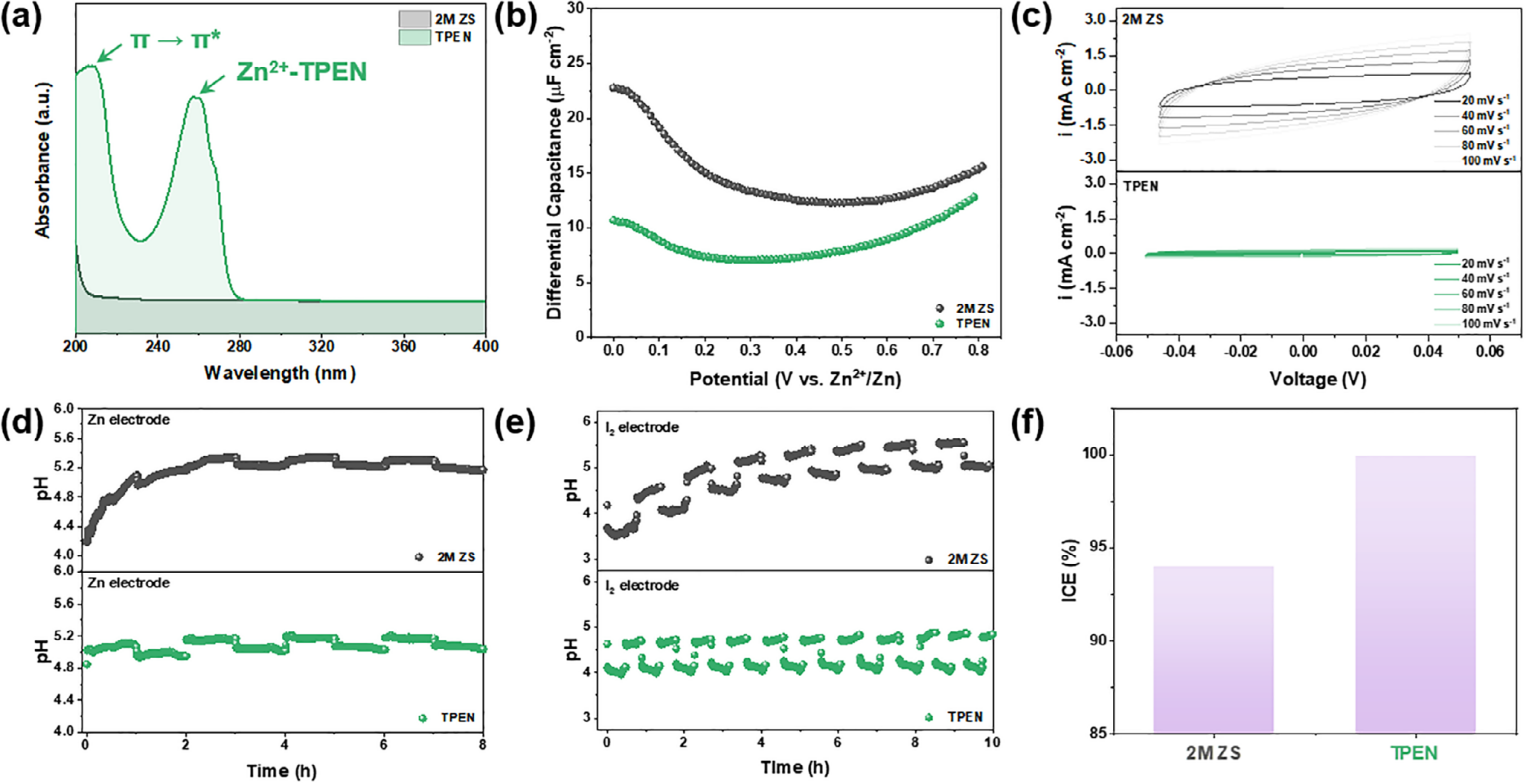
(a) UV–vis spectra of the 2 M ZS and TPEN electrolyte. (b) Differential capacitance measurement using 2 M ZS and TPEN electrolyte, respectively. (c) Cyclic voltammetry measurement recorded from I_2_@AC symmetric cells using 2 M ZS and TPEN electrolyte, respectively. (d) In‐situ pH measurement of Zn anode side in Zn||I2 cells using 2 M ZS and TPEN electrolytes. (e) In‐situ pH measurement of I2 cathode side in Zn||I_2_ cells using 2 M ZS and TPEN electrolytes. (f) Initial Coulombic efficiency (ICE) of Zn||I_2_ cells using 2 M ZS and TPEN electrolytes.

To verify if the TPEN chelating agent formed a chelating complex with Zn ions through electron donor–acceptor (EDA) interactions, UV‐vis spectroscopic analysis was conducted on a 2 M ZnSO4 (2 M ZS) electrolyte with TPEN (TPEN) (Figure 2a). Unlike the spectrum of the 2 M ZS electrolyte, the TPEN electrolyte displayed peaks at 260 nm and 210 nm, corresponding to the Zn^2^^+^‐TPEN chelating complex and the π → π* charge transfer in the pyridyl ring [40, 41]. This spectral feature supports the formation of Zn^2+^–TPEN coordination species through EDA interactions between TPEN and Zn^2+^. In addition to the spectroscopic analysis, DFT calculations were performed to evaluate the energetically favorable coordination geometry of the proposed Zn^2+^–TPEN complex. The calculation results suggest that the hexadentate configuration represents a thermodynamically favorable coordination model for the proposed Zn^2+^–TPEN complex (Figure S1). Together with the UV–vis results, these calculations provide complementary support for Zn^2+^–TPEN coordination, rather than a quantitative determination of solution‐phase speciation. To distinguish the coordination effects of Zn^2+^ from TPEN's intrinsic absorption or nonspecific ionic strength effects, additional UV–vis spectroscopic data were collected for TPEN dissolved in water, Na_2_SO_4_ solution, and a 2 M ZnSO_4_ electrolyte (Figure S2). The distinct absorption features observed in solutions containing ZnSO_4_ were either absent or significantly weaker in the water and Na_2_SO_4_ control groups, suggesting that the spectral changes do not occur simply due to the presence of sulfate ions or the aqueous ionic medium alone, but rather require the simultaneous presence of TPEN and Zn^2+^. Furthermore, as the TPEN concentration increased within the ZnSO_4_ electrolyte, the intensity of the characteristic absorption band increased systematically (Figure S3). This concentration‐dependent spectral change supports the formation of Zn^2+^ coordination species derived from TPEN in solution, rather than spectral features arising from undissolved TPEN or simple physical mixing. This was further supported by optical images of each electrolyte, showing only transparent solutions without undissolved powder in vials containing the Zn salt (Figure S4). The TPEN concentration was optimized to balance both the stability of the Zn anode and the polyiodide capturing ability. Zn||Zn symmetric cells containing 0.005, 0.01, and 0.05 wt.% TPEN exhibited the most stable long‐term cycling performance at 0.01 wt.%; the inferior stability at 0.005 wt.% indicates insufficient interfacial coverage, whereas the decreased stability at 0.05 wt.% suggests that excessive TCC accumulation may increase interfacial resistance or disturb Zn^2+^ transfer (Figure S5). Unlike the non‐monotonic dependence of Zn cycling stability, the polyiodide‐capturing rate increased with TPEN concentration, demonstrating that the optimal additive concentration must balance the distinct requirements of the two electrode interfaces (Figure S6).

### 2.2 EDL Reconstruction and pH Stabilization at the Zn Anode

To evaluate the effect of this TCC, an organic‐ligand‐based complex with a large cluster size, on the Zn metal interface, differential capacitance measurements were performed. These measurements showed that the TPEN electrolyte exhibited lower capacitance values than the 2 M ZS electrolyte (Figure 2b) [42, 43]. This lower capacitance in the TPEN electrolyte is attributed to the hydrophobic organic ligands of the TCC, allowing for closer positioning near the electrode interface due to repulsive forces on water molecules within the EDL. In addition, the large cluster size of the TCC reduces the concentration of Zn ions within the EDL. Furthermore, CA experiments indicated that the TPEN electrolyte showed a lower double‐layer charging current density than the 2 M ZS electrolyte, confirming slower charge accumulation near the interface and lower capacitance (Figure S7) [44, 45]. To investigate the adsorption behavior of TCC at the electrode interface, Raman spectroscopy was performed on the surface of the Zn metal cycled in the non‐Faradaic potential region. Peaks corresponding to C–H stretching vibrations appeared at 1450 and 2850 cm^−1^, while a peak corresponding to C–N stretching vibrations appeared at 1280 cm^−1^. These features were absent on Zn metal when the 2 M ZS electrolyte was used (Figure S8) [46, 47, 48, 49]. This observation confirms the presence of TCC at the Zn metal interface through adsorption interactions. To confirm if the restructuring of the EDL at the cathode interface also occurred at the anode interface, a symmetric cell with an iodine cathode was assembled, and CV was conducted in the non‐Faradaic potential region. The results indicated that in the electrolyte containing TCC, the double‐layer charging current was generally lower than that of the reference electrolyte, confirming a decrease in the effective capacitance at the interface (Figure 2c). These results indicate that TCC approaches and adsorbs near the iodine electrode interface, leading to modifications of the EDL and regulation of interfacial charge accumulation. Accordingly, the TPEN‐derived TCC exists near both the Zn anode and the I_2_ cathode, providing a basis for regulating the cathodic interfacial reaction environment. Moreover, pH stability is a critical factor affecting interfacial stability, as pH changes during the Zn plating/stripping process can lead to the formation of resistant ZHS byproducts and short circuits. In situ pH measurements revealed that the pH change (ΔpH = pH_final_ − pH_initial_ = 0.07) in the TPEN electrolyte during the plating/stripping process was significantly smaller than that in the 2 M ZS electrolyte (ΔpH = 0.18) (Figure 2d and Figure S9) [50]. This confirmed that TCC, present at the Zn metal interface via adsorption, can induce an inhibitory effect on side reactions, such as the HER and corrosion, by reducing the hydrophobicity and concentration of fully hydrated Zn ions. These findings suggest that TCC can suppress HER and corrosion by adsorbing onto the Zn metal interface and limiting the access of water and fully hydrated Zn^2^^+^ to the interface. Additionally, the suppression of side reactions may alleviate the localized alkalization accompanying the electrodeposition process, leading to interfacial pH stabilization. To investigate in greater detail whether this phenomenon is related to the proton‐accepting capacity of the nitrogen sites derived from TPEN, stepwise acid addition measurements were performed on the control electrolyte and the TPEN‐containing electrolyte (Figure S10). When the same amount of dilute H_2_SO_4_ was added, the electrolyte containing TPEN exhibited a smaller decrease in pH than the control electrolyte, indicating that the pyridyl and tertiary amine nitrogen sites can partially accept the added protons through protonation equilibrium. Therefore, when TPEN‐derived species are concentrated near the electrode through Zn^2+^ coordination and interfacial adsorption, their protonatable donor sites can locally mitigate the transient proton activity changes that occur during Zn plating/stripping and water reduction. Therefore, the observed pH stabilization appears to result from a combination of the suppression of the proton‐consuming HER and the localized protonation/deprotonation of TPEN‐derived species accumulated at the interface. This stabilization could potentially weaken the pathway through which crosstalk induces an increase in pH at the cathode interface, thereby promoting iodine hydrolysis, dissolution, and polyiodide formation. To validate this, in situ pH measurements were conducted at the iodine cathode. The results revealed that in the TCC electrolyte, the magnitude of the pH increase at the cathode interface during charging and discharging decreased compared to that in the 2 M ZS electrolyte, with more moderate fluctuations (Figure 2e). This demonstrates that TCC, in addition to its anode‐stabilizing effect, can mitigate interfacial chemical disturbances at the cathode, contributing to pH stabilization. This supports its association with the inhibition of iodine hydrolysis, dissolution, and polyiodide formation. Therefore, the simultaneous stabilization of the anode and cathode interfaces induced by TCC effectively weakened the degradation loop formed by the crosstalk between the electrodes. Consequently, this approach significantly improved the initial Coulombic efficiency of Zn–I_2_ batteries by reducing irreversible reactions during the initial cycle, a crucial parameter for ESS applications (Figure 2f).

### 2.3 Suppressed Side Reactions and Uniform Deposition Behavior of Zn Metal

**FIGURE 3:**
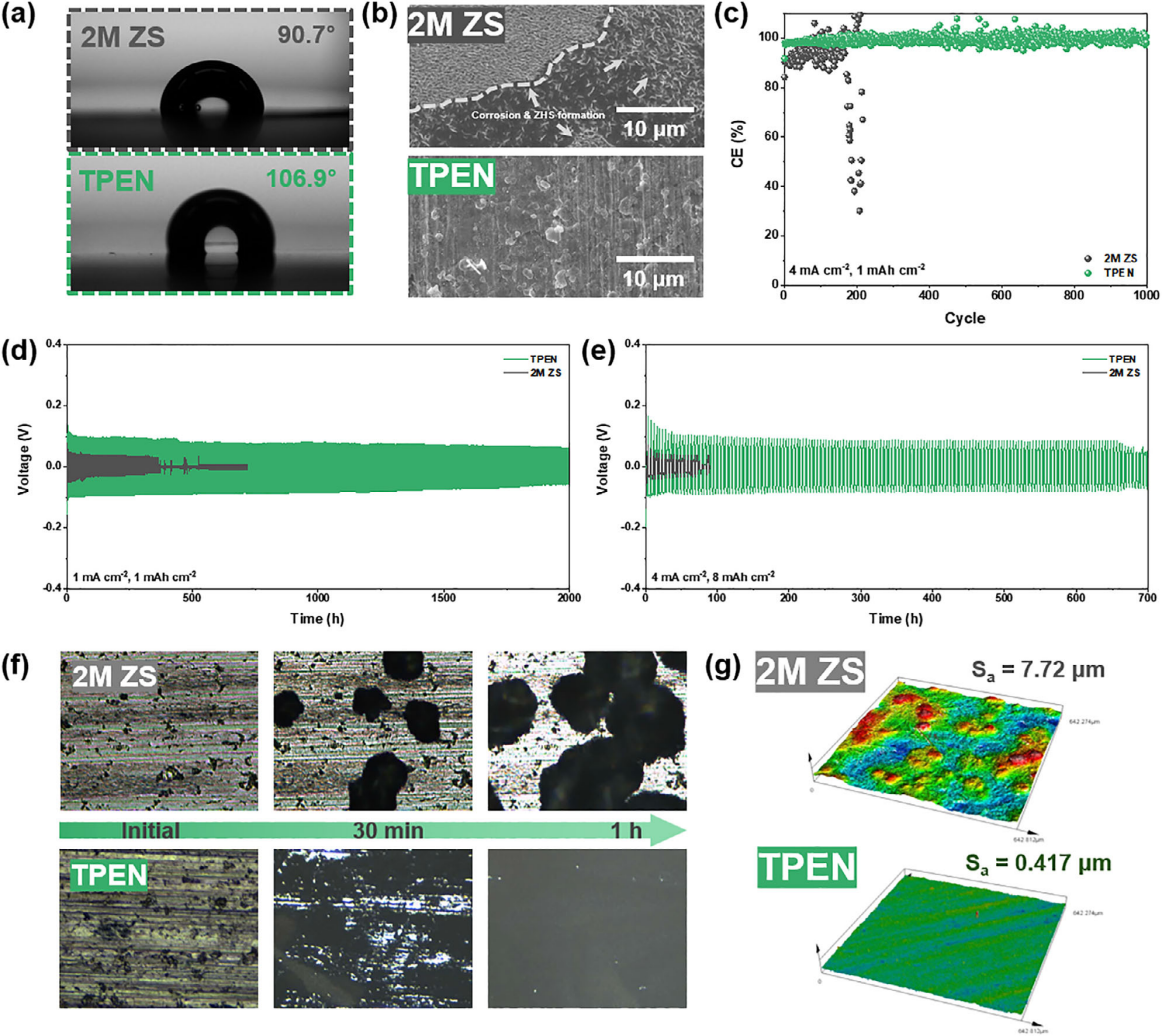
(a) Contact angle measurement using 2 M ZS with and without TPEN, respectively. (b) SEM images of Zn metal surface after 24 h immersion test. (c) Coulombic efficiency (CE) plots of Zn||Ti asymmetric cell using 2 M ZS and TPEN electrolyte. (d) Cycling performance of Zn||Zn symmetric cells at 1 mA cm^−2^ and 1 mAh cm^−2^ in 2 M ZS and TPEN electrolyte. (e) Cycling performance of Zn||Zn symmetric cells at 4 mA cm^−2^ and 8 mAh cm^−2^ in 2 M ZS and TPEN electrolyte. (f) In‐situ optical microscope (OM) images of Zn substrates during Zn plating and stripping at 3 mA cm^−2^ in the 2 M ZS and TPEN electrolyte. (g) 3D LCSM images of Zn metal deposition in 2 M ZS and TPEN electrolyte.

To investigate the effect of TPEN addition on the aqueous hydrogen‐bonding environment, we performed deconvolution analysis of the O‐H stretching region in the Raman spectrum (Figure S11). The broad O‐H band was separated into contributions associated with water clusters forming strong, moderate, and relatively weak hydrogen bonds. Compared to the 2 M ZnSO_4_ electrolyte, the TPEN electrolyte exhibited a lower relative contribution from strongly hydrogen‐bonded water and a higher contribution from moderately or weakly hydrogen‐bonded components. This redistribution suggests that TPEN‐derived coordination species disrupt the extended water hydrogen‐bonding network. Therefore, when considered in conjunction with interfacial adsorption and wettability measurements, these findings support an electrolyte and interfacial environment characterized by reduced accessibility and collective organization of reactive water near Zn. The contact angle increased in the presence of TCC at the electrolyte‐electrode interface during measurement, indicating improved hydrophobicity at the Zn metal interface. This enhancement plays a crucial role in reducing cathodic side reactions such as the HER and corrosion by restricting water access and hydrated species involvement (Figure 3a). The effect of this interfacial environment on water reduction was further evaluated using linear sweep voltammetry with a Zn||Ti cell (Figure S12). Compared to the 2 M ZS electrolyte, the TPEN electrolyte exhibited a delayed increase in cathodic current and a higher apparent hydrogen evolution reaction (HER) overpotential, suggesting that the hydrogen evolution reaction was kinetically inhibited. This result is consistent with a lower probability of reactive water reaching active Zn sites when TCC‐derived species occupy the interface. The suppressed HER kinetics also provides a mechanistic basis for the smaller local pH variation. To verify whether the decrease in water‐related reactivity observed in the experiment is thermodynamically consistent with competitive interfacial occupation, DFT calculations were performed on the adsorption of H_2_O and TCC on a representative Zn surface (Figure S13). TCC exhibited significantly lower adsorption properties than H_2_O molecules on the Zn surface, suggesting that the chelate complex can compete with water to preferentially occupy sites on the Zn surface. Consistent preference for TCC adsorption provides molecular‐level evidence that the results from Raman spectroscopy, contact angle, and HER measurements can be interpreted as a consequence of reduced water accessibility at the Zn interface. These effects collectively enhanced the efficiency and stability of the Zn metal anode with TPEN. To analyze the suppression of side reactions involving water molecules through TCC adsorption at the Zn metal interface, the Zn metal surface was examined after 24 h of electrolyte immersion. In the 2 M ZS electrolyte, sporadic formation of flake‐like ZHS compounds occurred, while the TPEN electrolyte prevented byproduct formation, maintaining a clean surface (Figure 3b). XRD analysis further validated the reduction in byproduct formation with the TPEN additive. In the 2 M ZS electrolyte, a distinct ZHS‐specific diffraction peak appeared at 2θ ≈ 12.4°, which was significantly diminished in intensity in the TPEN electrolyte (Figure S14) [51, 52, 53].

The Zn||Ti battery with a 2 M ZS electrolyte initially exhibited a CE of 84.28% at a current density of 4 mA cm^−2^ and an areal capacity of 1 mAh cm^−2^, failing within fewer than 200 cycles. In contrast, the Zn||Ti battery with TPEN electrolyte demonstrated an improved initial CE of 91.82% at the same current density and specific capacity, enabling stable cycling for over 1000 cycles with a CE of 99.67% (Figure 3c). To address the significance of low C‐rate performance in ESS applications, a Zn||Ti asymmetric battery was tested at 0.25 C. Despite severe test conditions leading to notable side reactions at a current density of 0.25 mA cm^−2^ and an areal capacity of 1 mAh cm^−2^ (0.25 C), the Zn||Ti battery with 2 M ZS electrolyte experienced performance degradation in less than 10 cycles. Conversely, the Zn||Ti battery utilizing TPEN electrolyte cycled steadily for over 100 cycles while maintaining a high Coulombic efficiency of 98.45% (Figure S15). Therefore, interfacial engineering through TCC, characterized by hydrophobicity and a large cluster size, proves advantageous in suppressing side reactions and enhancing the reversibility of zinc deposition.

The Zn||Zn symmetric cells were constructed to assess the reversibility and cycling stability of the Zn cathode in different electrolytes. Figure 3d and **e** show that the addition of TPEN enables stable cycling performance and reversibility of the zinc cathode. In TPEN electrolyte, the Zn||Zn battery demonstrated a cycle life of 2000 h at 1 mA cm^−2^ and 1 mAh cm^−2^, approximately 5.5 times longer than that in 2 M ZS electrolyte (370 h) (Figure 3d). Even under high plating capacity of 8 mAh cm^−2^, corresponding to a Zn utilization rate of 27.6%, it exhibited excellent stability of 700 h compared to the cell using 2 M ZS electrolyte (83 h) (Figure 3e). Additionally, the rate performance of the Zn||Zn cell in TPEN electrolyte showed a more consistent voltage profile at different current densities (Figure S16). The time‐voltage profile, showing increased voltage polarization and stable voltage hysteresis, also indicates the reversible and stable zinc plating/stripping processes in the TPEN electrolyte. This highlights that TPEN‐containing electrolytes can effectively inhibit side reactions and dendrite growth at high zinc utilization rates.

Optical microscopy was used to confirm the observed effects. The 2 M ZS electrolyte exhibited the presence of aggregated coarse and irregular zinc clusters, along with insulating byproducts. In contrast, the TPEN electrolyte maintained a consistently uniform, smooth, and dense deposition layer even after 1 h of plating (Figure 3f). The synergistic effects of the adsorbed protective layer, pH buffering, and adequate overpotential collectively promoted a uniform zinc plating and stripping process. Laser confocal scanning microscopy (LCSM) analysis revealed that zinc deposited in the 2 M ZS electrolyte exhibited a rough surface morphology (S_a_ = 7.72), while the TPEN electrolyte resulted in a significantly smoother surface (S_a_ = 0.417) (Figure 3g). This quantitative reduction in surface roughness highlights the effective modulation of zinc deposition behavior by TCC through synergistic interfacial mechanisms, leading to dense, flat zinc growth without dendritic crystals.

The adsorption behavior of TCC, its pH‐stabilizing capacity, and its ability to induce a high surface overpotential were evident in the structural changes during the Zn plating/stripping process. XRD analysis showed that zinc deposited in a 2 M ZS electrolyte displayed anisotropic growth with coexisting (002) and (101) planes (I_002_/I_101_ = 0.61 after 8 mAh cm^−^^2^ deposition), indicating no preferred orientation. In contrast, the TPEN electrolyte increased the I_002_/I_101_ ratio to 0.88, promoting a (002) plane‐dominant growth trend (Figure S17). To verify whether preferred orientation appears not only in the final deposition state but also gradually over time, additional XRD patterns were collected as the Zn deposition capacity increased and analyzed using the relative texture coefficient (RTC) (Figure S18). In the TPEN electrolyte, the RTC of the Zn (002) plane increased with deposition capacity, whereas in the 2 M ZS electrolyte, the contribution of the (002) orientation tended to decrease as deposition progressed. These capacity‐dependent changes demonstrate that TCC does not merely modify the initial substrate surface but continuously influences crystal growth throughout the entire Zn deposition process. Facet‐dependent DFT calculations provide a thermodynamic basis for these changes (Figure S13). Among the Zn surfaces investigated, TCC exhibited the most favorable adsorption properties on the Zn (002) face. This selective adsorption can alter the relative interfacial energy and growth kinetics of the exposed faces, thereby influencing which crystal faces remain exposed during the electrodeposition process. Therefore, the calculated adsorption tendency is consistent with the increase in the (002) texture coefficient observed experimentally. Selective adsorption of TCC partially blocks heterogeneous sites on the Zn surface, thereby increasing the nucleation overpotential and, as a result, promoting the formation of smaller, more uniformly distributed Zn nuclei based on the classical nucleation theory. This phenomenon is consistent with enhanced Zn (002) texture and a homogeneous deposition morphology.

These findings were further confirmed through SEM after plating at 8 mAh cm^−2^. The 2 M ZS electrolyte resulted in a coarse, chunk‐like, and non‐uniform Zn deposit, while the TPEN electrolyte facilitated dense and uniform Zn growth (Figure S19). This morphological enhancement suggests that the TCC formed by TPEN addition exists in an adsorbed state at the Zn metal interface, creating an interfacial environment with reduced water accessibility. This condition suppresses water‐induced side reactions by providing additional surface overpotential at the interface.

### 2.4 Polyiodide Binding and Interfacial Reaction Regulation at the I2 Cathode

**FIGURE 4:**
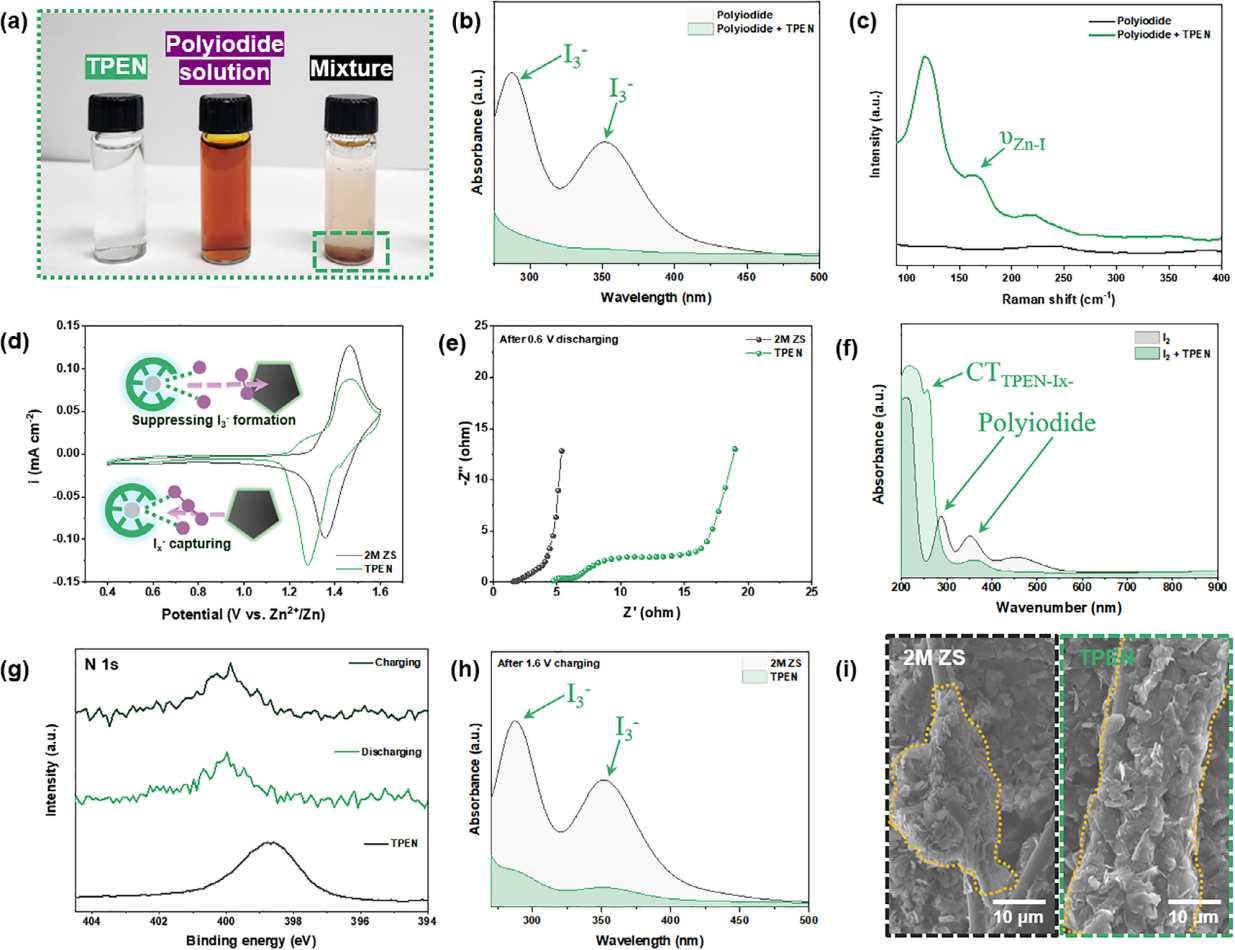
(a) Photographs for the formation of the TCC‐I_x_^−^ precipitate. (b) UV–vis spectra of the supernatant. (c) Raman spectra of solid precipitate powder. (d) Cyclic voltammetry (CV) curves for Zn||I2 batteries in 2 M ZS and TPEN electrolytes. (e) Nyquist plots for Zn||I_2_ batteries in 2 M ZS and TPEN electrolyte. (f) UV–vis spectra of Iodine solution with and without TPEN electrolyte. (g) N 1s XPS spectra of TPEN powder, positive electrodes discharged to 0.6 V and charged to 1.6 V using TPEN electrolyte. (h) UV–vis spectra of electrolyte after charging to 1.6 V. (i) SEM images of positive electrodes charged to 1.6 V using 2 M ZS and TPEN electrolytes.

To investigate the binding characteristics between the TCC and polyiodide ions and to verify the shuttle effect inhibition, precipitation phenomena were observed by adding TPEN electrolyte to ZnI_3_ (ZnI_2_ + I_2_) solutions. Upon adding 2 M ZS electrolyte to the deep orange I_3_^−^ solution, no significant color change was observed (Figure S20). However, upon adding TPEN to the I_3_^−^ solution, distinct layer separation occurred: a solid precipitate formed at the vial bottom, and the upper layer's color significantly lightened (Figure 4a). This suggests the formation of a solid complex between TPEN and I_3_^−^. UV–vis spectroscopy was performed on the supernatant to confirm the binding between TPEN and I_3_^−^ (Figure 4b). In the UV–vis spectrum, the peaks at 290 and 350 nm correspond to I_3_^−^. The signal intensity of I_3_^−^ decreased significantly after TPEN addition, indicating the strong binding effect of TCC on the polyiodide ion [54, 55]. Raman spectroscopy analysis was conducted to confirm the chemical composition of the formed solid precipitate (Figure 4c). A peak at 167.1 cm^−1^ corresponding to the Zn‐I asymmetric stretching vibration was detected in the Raman spectrum of the precipitate, suggesting a solid compound formed due to the binding interaction between Zn^2^^+^ and iodide ions (I_x_^−^) [56, 57]. Raman spectroscopic analysis was performed on the TPEN powder and compared with the solid precipitate to confirm that the precipitate formation resulted from the interactions between TCC and iodide ions (Figure S21). A peak at 1000 cm^−1^ corresponding to the pyridine ring breathing vibration was observed. In addition, the peak at 1600 cm^−1^ attributed to the C = C/C = N stretching vibrations exhibited a blue shift. A red shift was also observed for the peak at 1230 cm^−1^ corresponding to the C─N/C─H bonding vibrations [48, 58, 59, 60]. Furthermore, to verify that the capture of I_x_^−^ species stems from TCC formation, Raman spectroscopic analysis was performed on NaI_x_ solutions containing identical concentrations of 2 M ZS, TPEN, and NaSO_4_ with TPEN electrolytes. A higher intensity of the peak at 167.1 cm^−1^, corresponding to the Zn–I^+^ bonding vibration, was clearly observed in the sample containing TCC [56, 57]. This result demonstrates the strong binding capability of TCC toward I_x_^−^ species, which arises from the EDA interaction between Zn^2^^+^ and TPEN (Figure S22). To compare the intrinsic affinity of the relevant electrolyte species toward polyiodide, DFT binding energies were calculated for I_3_^−^ interacting with H_2_O, a hydrated Zn^2+^ cluster, TPEN, and TCC (Figure S23). The interactions with TPEN and TCC were more energetically favorable than those with H_2_O or hydrated Zn^2+^, demonstrating that the nitrogen‐rich chelating framework provides a preferential association environment for polyiodide. In particular, the TCC–I_3_^−^ interaction indicates that Zn^2+^ coordination does not eliminate the polyiodide affinity of TPEN; instead, the chelated structure retains the ability to associate with iodine species through a combination of electrostatic polarization, donor‐acceptor interaction, and spatial confinement around the multidentate ligand framework. These calculations provide a thermodynamic basis for the reduced polyiodide absorption intensity and precipitate formation observed experimentally. The dynamic capture behavior was further evaluated by monitoring the polyiodide absorption of the supernatant as a function of time after addition of either the 2 M ZS electrolyte or the TPEN electrolyte (Figure S24). Under otherwise identical conditions, the characteristic polyiodide absorption decreased more rapidly in the presence of TCC. This accelerated decrease indicates that the effect of TCC is not limited to favorable final‐state binding energy, but also enables efficient removal of freely dissolved polyiodide from the solution phase on an experimentally relevant timescale. The agreement between the calculated binding affinity and the time‐dependent spectroscopic response supports a mechanism in which TCC reduces the free polyiodide concentration available for long‐range diffusion and shuttle reactions. To determine whether the TCC–iodine interaction persists during actual electrochemical operation, Raman spectra were collected from the electrolyte/electrode region near the I_2_ cathode at selected states of charge (Figure S25). The Raman feature assigned to Zn‐I bonding decreased systematically with the cell discharging, indicating reversible evolution of TCC‐associated iodine species. The state‐dependent response differs from a static precipitation phenomenon and suggests that the TCC–polyiodide interaction participates in the dynamic conversion of iodine species at the cathode interface. These observations support a mechanism in which TCC temporarily associates with iodine intermediates, decreases their residence in the free solution phase, and facilitates their conversion near the electrode interface.

### 2.5 Inhibited Shuttle Effects and Stabilized I_2_ Redox Kinetics

This validates the ability of the chelating complex to bind and capture iodide ions. To assess the impact of the chelating complex in the electrolyte on the electrochemical iodine redox reaction, CV tests were conducted using a Zn||I_2_ cell (Figure 4d). In comparison to the cell with the 2 M ZS electrolyte, which exhibited a reduction peak at 1.38 V, the cell using the TPEN electrolyte showed a reduction peak at 1.29 V. This outcome is attributed to the presence of the TPEN‐derived chelating complex at the electrode interface, which diminishes the concentration of hydrated Zn ions. Regarding the oxidation reaction, a novel oxidation peak is evident at 1.31 V in the cell employing the TPEN electrolyte. This suggests that the chelating complex‐iodide compound, generated in the solid phase through the reduction (discharge) reaction, initially partakes in the oxidation reaction due to its proximity to the electrode surface. The existence of this solid‐phase deposit post the reduction reaction can be verified through EIS analysis (Figure 4e). In the fully discharged state (0.6 V) of a Zn||I_2_ cell utilizing TPEN electrolyte, a semicircle in the mid‐frequency region was observed, contrasting with the absence of this feature in the cell using 2 M ZS electrolyte. This suggests the development of a new interface due to the solid‐phase layer on the electrode surface. Moreover, post‐cycling UV–vis analysis showed that the characteristic TPEN/TCC absorption features remained detectable after 5, 15, and 30 cycles (Figure S26). This indicates that TPEN/TCC‐derived species persist during cycling and are not rapidly and completely consumed during the initial cycles, although these measurements do not establish the complete molecular integrity of TPEN or exclude minor decomposition pathways.

To investigate the impact of the TPEN chelating complex on the I_2_ deposition‐based charging (oxidation) process, UV–vis spectroscopic analysis was conducted on I_2_ solutions with and without TPEN (Figure 4f). The UV–vis spectrum of the pure I_2_ solution displayed peaks at 290 and 350 nm corresponding to I_3_^−^, and a peak at 450 nm corresponding to I_2_ [54, 55]. In contrast, the TPEN‐containing sample exhibited reduced peak intensities for I_3_^−^ and I_2_, along with a new peak at 260 nm corresponding to the formation of the TPEN‐I_2_ (I_3_^−^) charge‐transfer complex [61, 62]. This suggests that the TCC interacts with deposited iodine through Lewis acid‐base interactions, present in an adsorbed state on the iodine surface. Consequently, the TPEN‐derived chelating complex can engage in the I^−^/I_2_ electrochemical redox reaction near the electrode interface by interacting with iodide and iodine. This interaction was further confirmed through post‐mortem XPS analysis of discharged and charged electrode samples (Figure 4g). Comparison of the high‐resolution N 1s XPS spectra of TPEN powder and discharged/charged electrode samples revealed that the pristine powder exhibited a peak at 398.8 eV, whereas the electrode samples showed a peak at 400.1 eV, corresponding to a higher binding energy [63, 64]. These results confirm the presence of the TPEN chelating complex near the electrode interface, where it is actively involved in the charging and discharging processes. The TCC at the interface participates in redox reactions through binding interactions with redox species, thereby suppressing the migration of polyiodide species that readily diffuse through the bulk electrolyte. This behavior facilitates rapid mass transfer during electrochemical reactions. UV–vis spectroscopic analysis of the electrolyte post‐charging process in Zn||I_2_ batteries revealed a notable reduction in the intensity of I_3_^−^ associated peaks (290 nm and 350 nm) in cells using the TPEN electrolyte compared to those with the 2 M ZS electrolyte (Figure 4h). This observation confirmed the suppression of polyiodide migration by chelating complexes. Furthermore, CV analysis conducted at different scan rates demonstrated a Randles–Sevcik relationship. Cells employing TPEN electrolyte exhibited a steeper slope and enhanced linearity in the peak current‐scan rate^1/2^ relationship in the diffusion‐controlled region (Figure S27) [65, 66]. The introduction of TPEN naturally leads to the formation of a TPEN chelating complex within the electrolyte, effectively binding to iodide/polyiodide through Lewis acid‐base and electrostatic interactions. This binding hinders the excessive dissolution of I_2_, as well as the formation and diffusion of free I_3_^−^ during the charging/discharging process. This chemical stabilization reduces the i_p_‐v^1/2^ slope based on the Randles‐Sevcik equation of the TPEN electrolyte due to the decrease in reactive free polyiodide concentration and the effective reduction in diffusion rate. However, the peak potential remained nearly stationary despite changes in the scan rate. This suggests that TCC stabilizes the electrode‐electrolyte interface, suppresses instantaneous changes in the polyiodide concentration, and maintains a constant distribution of redox species during charging, thereby enhancing the reversibility of the reaction.

To verify the effect of the TPEN‐derived chelating complex on the electrodeposition process, SEM analysis was conducted on the I_2_ electrode in the charged state of the Zn–I_2_ battery with 2 M ZS and TPEN electrolytes (Figure 4i). In contrast to the uneven and lumpy deposit observed on the electrode in the cell with the 2 M ZS electrolyte, the electrode in the TPEN electrolyte cell displayed a more uniform and conformal deposition. This difference is attributed to the influence of the chelating complex.

### 2.6 Direct Evidence of Crosstalk Suppression via In Situ Analysis

**FIGURE 5:**
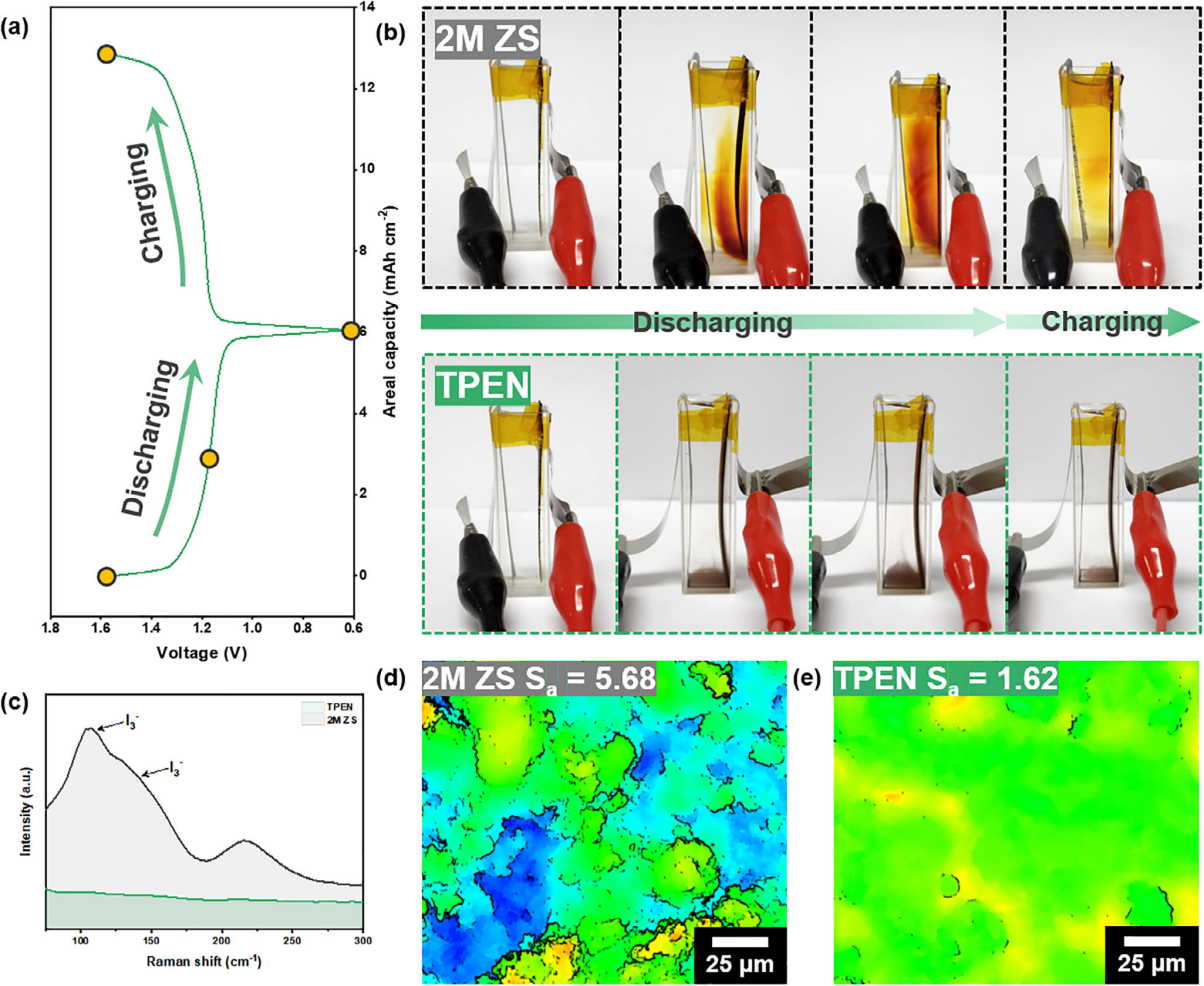
(a) GCD curves with marked voltage points of Zn||I_2_ batteries using 2 M ZS and TPEN electrolytes. (b) Corresponding in situ optical photographs of the shuttle effect in different batteries. (c) Post‐mortem analysis of Raman spectra of Zn metal after charging to 1.6 V using 2 M ZS and TPEN electrolyte. 3D LCSM images of Zn metal electrode from Zn||I_2_ batteries using (d) 2 M ZS and (e) TPEN electrolyte.

The low coulombic efficiency and self‐discharge in Zn–I_2_ batteries are due to the shuttling effect of polyiodide intermediates. To elucidate this effect, we conducted in situ optical microscopy at various voltages for two electrolytes (Figure 5a,b). In the cell using the 2 M ZS electrolyte, a noticeable yellow color appears at 1.2 V and deepens at 0.6 V, indicating the emergence and accumulation of polyiodide. As the cell charges, the yellow color fades gradually but diffuses into the adjacent solution due to polyiodide diffusion driven by the concentration gradient. In contrast, cells using the TPEN electrolyte show a significant reduction in color changes throughout the entire charge/discharge cycle, indicating suppression of the shuttling effect. Furthermore, Raman spectroscopic analysis of the Zn metal electrode surface in fully charged cells reveals a significant reduction in the intensity of peaks corresponding to I_3_^−^ (110 and 144.6 cm^−1^) compared to cells using the 2 M ZS electrolyte (Figure 5c) [67]. This observation confirms that the TPEN chelating complex plays a key role in suppressing polyiodide diffusion. Furthermore, the corrosion of the Zn metal surface caused by self‐discharge from highly oxidized, shuttling polyiodide species was evaluated using LCSM. Zinc cycled in 2 M ZS electrolyte exhibited a rough surface morphology (S_a_ = 5.68), while the TPEN electrolyte produces a smoother surface (S_a_ = 1.62) (Figure 5d,e). This reduction in surface roughness results from the inhibition of polyiodide shuttle‐driven corrosion by the TPEN‐derived chelating complex, which binds polyiodide intermediates effectively and acts as a diffusion barrier due to its bulky cluster size.

### 2.7 Improved Full Cell Performance Under High Loading Conditions

**FIGURE 6:**
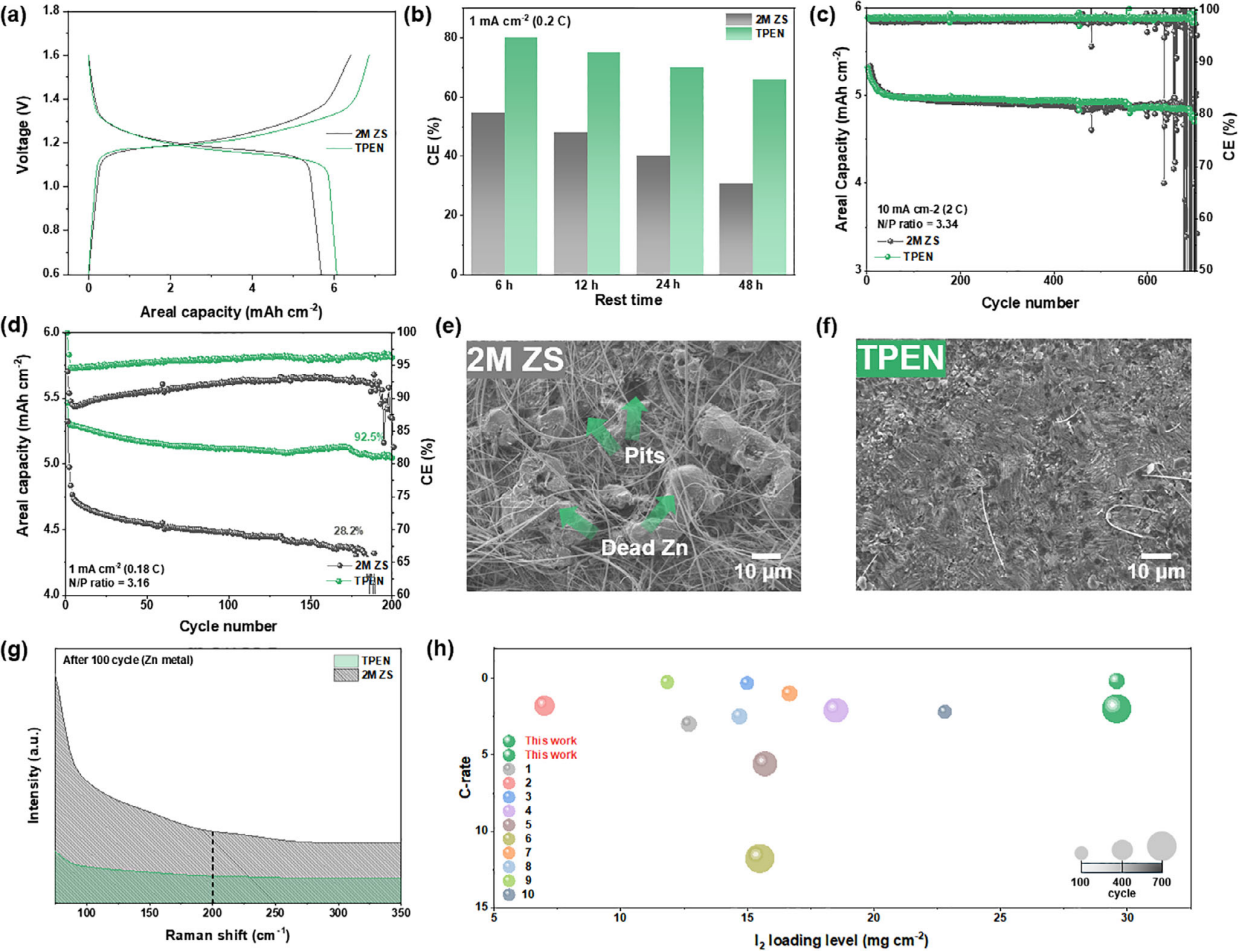
(a) Galvanostatic charge/discharge (GCD) curves for Zn||I_2_ batteries in 2 M ZS and TPEN electrolyte. (b) Self‐discharge performance batteries using 2 M ZS and TPEN electrolyte at 1 mA cm^−2^ (0.2 C). (c) Cycling performance at 10 mA cm^−2^ of high‐loading Zn||I_2_ batteries using 2 M ZS and TPEN electrolyte. (d) Cycling performance at 1 mA cm^−2^ of high‐loading Zn||I_2_ batteries using 2 M ZS and TPEN electrolyte. Post‐mortem analysis of SEM images of Zn metal after 100 cycles of high‐loading Zn||I_2_ batteries using (e) 2 M ZS and (f) TPEN electrolyte. (g) Post‐mortem analysis of Raman spectra of Zn metal after 100 cycles of high‐loading Zn||I_2_ batteries using 2 M ZS and TPEN electrolyte. (h) Comparison of testing conditions, including I_2_ loading mass and C‐rate, of Zn–I_2_ batteries with reported works.

In the galvanostatic charging and discharging experiment of Zn–I_2_ batteries, the TPEN electrolyte exhibited a specific capacity of 6.07 mAh cm^−^^2^, outperforming the 2 M ZS electrolyte (4.8 mAh cm^−^^2^) (Figure 6a). The self‐discharge test at 0.2 C‐rate in Figure 6b reveals that the battery with the 2 M ZS electrolyte retained 54.52%, 48.01%, 40.19%, and 30.61% of its capacity after 6, 12, 24, and 48 h of rest, respectively. In contrast, the TPEN electrolyte maintained a higher capacity retention rate of 65.89% even after 48 h of rest, showcasing superior self‐discharge prevention performance.

The superiority of TPEN is also evident under quasi‐open‐circuit voltage conditions (Figure S28). During charging and storage, the 2 M ZS electrolyte displayed a potential drop exceeding tens of millivolts, while the TPEN electrolyte showed only a drop of a few millivolts, indicating that TCC suppressed iodine dissolution and polyiodide (I_3_^−^) formation. Long‐term cycling tests were conducted using 2 M ZS and TPEN electrolytes to evaluate the electrochemical performance of the Zn||I_2_ battery with a high‐loading I_2_ cathode. As shown in Figure 6c, the Zn||I_2_ battery utilizing the 2 M ZS electrolyte exhibited a capacity retention of 65.73% after 700 cycles at 10 mA cm^−^^2^ (2 C) with a lower coulombic efficiency (CE) of 44.73% due to side reactions such as the shuttle effect and corrosion. These processes ultimately led to premature cell failure. In contrast, the battery using the TPEN electrolyte exhibited a higher capacity retention rate of 88.35% with an increased CE of 98.86%, attributed to the interfacial stabilization by TCC. The incorporation of TPEN significantly impacted the long‐term cycling stability of the Zn–I_2_ battery under low C‐rate test conditions, where iodine dissolution and polyiodide shuttling were intensified, and the TPEN electrolyte displayed excellent capacity retention characteristics. After 200 cycles at 1 mA cm^−^^2^ (0.18 C), the 2 M ZS electrolyte retained 28.2% of its capacity due to severe side reactions at both the cathode and anode interfaces, while the TPEN electrolyte retained 92.5% of its capacity (Figure 6d). As shown in Figure 6e,f, following 100 cycles, the Zn metal electrode in the Zn–I_2_ battery using the TPEN electrolyte exhibited a dense and uniform morphology with a smooth surface free of dendritic protrusions. In contrast, the Zn metal in batteries using the 2 M ZS electrolyte showed pronounced surface irregularities under the same cycling conditions. Under these conditions, severe pits, dead Zn, and a highly rough and non‐uniform surface were observed.

Post‐mortem analysis of Zn metal after cycling was conducted using Raman spectroscopy to investigate the effect of polyiodide migration during cycling (Figure 6g). A high‐intensity polyiodide‐related peak was observed on the electrode of the cell with a 2 M ZS electrolyte, ranging from 100 to 250 cm^−1^ [68]. In contrast, no polyiodide‐related signals were detected on the surface when an electrolyte containing TCC was utilized. These findings indicate that TCC effectively suppresses self‐discharge at the interface by inhibiting iodine dissolution and polyiodide migration. Raman spectroscopic analysis of the I_2_ positive electrode after 100 cycles for cells using 2 M ZS and TPEN electrolytes revealed peaks at 280 and 360 cm^−1^, corresponding to Zn–N stretching vibrations and C–N deformation, respectively (Figure S29) [69, 70, 71]. This suggests that TPEN/TCC‐derived species remain present near the positive electrode interface after prolonged cycling and may continue to participate in interfacial iodine redox processes. Lewis acid–base and electrostatic‐force‐mediated binding interactions contribute to polyiodide confinement and shuttling suppression. The thermal robustness of the TPEN‐based interfacial regulation was further evaluated using Zn||I_2_ full cells at 45°C (Figure S30). Under elevated‐temperature cycling at 5 mA cm^−2^, the cell using TPEN electrolyte maintained higher capacity retention and Coulombic efficiency than the cell using the 2 M ZS electrolyte. Because elevated temperature accelerates Zn corrosion, HER, and polyiodide diffusion, the improved stability indicates that the TCC‐mediated dual‐interface regulation remains electrochemically effective under thermally accelerated degradation conditions. Additionally, the practical feasibility of the TPEN electrolyte was evaluated in Zn||I_2_ pouch cells using a Zn foil thickness of 20 µm, an iodine loading of 29.58 mg cm^−2^, an E/C ratio of 6 g Ah^−1^, and an N/P ratio of 1.93 (Figure S31). The pouch cell using TPEN electrolyte retained its capacity after 40 cycles with an average Coulombic efficiency of 96.84%, outperforming the 2 M ZS electrolyte (sudden death after 14 cycle and Coulombic efficiency of 84.73%). These results demonstrate that TCC‐mediated dual‐interface regulation remains effective under practically constrained Zn and electrolyte conditions. Furthermore, three independently assembled pouch cells using TPEN electrolyte exhibited consistent areal capacity retention and Coulombic efficiency profiles under identical conditions, confirming the cell‐to‐cell reproducibility of the practical pouch‐cell performance (Figure S31).

To enhance the comparability of high‐capacity Zn–I_2_ battery performance, this study included a comparative analysis of iodine anode mass loading (mg cm^−2^), C‐rate, and cycle stability relative to existing literature (Figure 6h; Tables S1 and S2) [31, 65, 72, 73, 74, 75, 76, 77, 78, 79, 80, 81, 82, 83, 84]. Table S1 summarizes the electrochemical performance of previously reported Zn–I_2_ batteries, whereas Table S2 details the practical pouch‐type cell parameters used in this work, including additive concentration, areal capacity, Zn thickness, N/P ratio, and current density. The analysis confirmed that this study conducted low C‐rate long‐term cycling under high I_2_ loading conditions compared to previous studies, validating performance under conditions resembling high‐energy‐density ESS operation. Notably, even under these rigorous conditions, the incorporation of TCC significantly enhanced long‐term stability by stabilizing both the anode and cathode interfaces and reducing crosstalk.

### 2.8 Dual‐Interface Stabilization Mechanism Enabled by Chelation

In this study, we propose a chelation‐driven dual‐interface regulation strategy by introducing TPEN as an electrolyte additive. This approach addresses the bottleneck arising from the coupled HER and corrosion processes and the polyiodide shuttle, whose interfacial crosstalk simultaneously degrades the lifespan and efficiency of aqueous Zn–I_2_ batteries (Figure S32). TPEN coordinates with Zn^2+^ to form Zn^2+^–TPEN coordination species, represented here as TCC, whose interfacial adsorption contributes to regulation of the anode and cathode interfacial environments. At the Zn anode, the TCC modulates the EDL into an interfacial environment with reduced water accessibility and induces a localized overpotential, promoting homogeneous nucleation, suppressing the HER and corrosion, and enabling uniform and dense deposition. Furthermore, protonatable donor sites of the TPEN buffer pH fluctuations inhibit ZHS formation and enhance reversibility, even under high Zn utilization conditions. At the I_2_ cathode, TCC binds and traps polyiodide through Lewis acid–base interactions with iodide/polyiodide, reducing the concentration of free shuttle species and mitigating self‐discharge and shuttling. It localizes interfacial reactions, decreases concentration polarization, and enhances the uniformity of I_2_ deposition/dissolution, thereby improving redox reversibility. Therefore, the TCC experimentally demonstrated the ability to simultaneously regulate both the anode and cathode interfacial reactions with a single additive, presenting an integrated interfacial design platform to suppress crosstalk‐induced degradation in aqueous metal–halide series batteries.

## 3 Conclusions

This study identified a degradation loop in which the anode HER, corrosion, and anode polyiodide shuttle mutually amplify each other through crosstalk, representing the primary bottleneck in aqueous Zn–I_2_ batteries. The study proposed a TPEN‐based chelation‐driven dual‐interface regulation strategy. The spectroscopic and computational results support the formation of Zn^2+^–TPEN coordination species (TCC) in the electrolyte. The interfacial adsorption of this chelate simultaneously reconfigures the anode and cathode interfacial environments. At the Zn anode, TCC induces an interfacial environment with reduced water accessibility, suppressing the HER and corrosion while promoting uniform deposition. Protonatable donor sites of TPEN further buffer local pH fluctuations and inhibit ZHS formation. At the I_2_ cathode, TCC traps polyiodide, reduces shuttling and self‐discharge, and enhances I_2_ deposition uniformity by localizing the reactions near the interface. Consequently, a Zn||Zn symmetric cell operated for over 2000 h, and a Zn–I_2_ cell under high I_2_ loading conditions achieved a CE of over 96.2% and a 92.5% capacity retention rate after 200 cycles. This work provides a chelation‐mediated molecular design strategy for dual‐interface regulation in aqueous Zn–I_2_ batteries. Although demonstrated here for Zn–I_2_ chemistry, this molecular design principle may provide useful guidance for the development of multifunctional electrolyte additives in other aqueous Zn battery systems, subject to further experimental validation.

## 4 Experimental Methods

### 4.1 Materials

ZnSO_4_·7H_2_O was acquired from Junsei Chemical Co. TPEN was obtained from TCI Chemicals. Sodium iodide (99.0%), zinc iodide (98.0%), and iodine (99.0%) were also procured from TCI Chemicals. Active carbon (YP‐50F) was sourced from Kuraray.

### 4.2 Preparation of Electrolyte

The blank electrolyte of 2 M ZnSO_4_ was created by dissolving ZnSO_4_·7H_2_O in deionized water. To prepare the electrolyte with additives, TPEN was then added to the initial blank electrolyte to make the 2 M ZnSO_4_ with 0.005, 0.01, and 0.05 wt.% TPEN electrolyte. The optimized concentration of TPEN was 0.01 wt.%.

### 4.3 Preparation of Positive Electrode

First, an activated carbon (AC) dry electrode was created using a mixture of AC, conductive carbon (Super C, TIMCAL), and polytetrafluoroethylene in a ratio of 70:25:5. The kneaded mixture was roll‐pressed to form the dry electrode. These electrodes were then placed in a glass vial with a specific quantity of I_2_ powder and thermally treated at 110°C for 12 h to fabricate the I_2_@AC positive electrodes. The loading of I_2_ in the I_2_@AC positive electrodes was adjusted by varying the amount of I_2_ used, and the I_2_ content of the electrodes was determined by measuring the change in electrode mass before and after I_2_ loading.

### 4.4 Material Characterization

Fourier‐transform infrared (FT‐IR) spectra were recorded with an FT‐IR 4700s (JASCO) instrument equipped with an attenuated total reflection accessory. Raman spectra were obtained using a confocal Raman spectrometer (NT‐MDT) with an excitation wavelength of 532 nm. The pH of the electrolyte was measured with a real‐time pH meter (Thermo Fisher Scientific). Ultraviolet–visible (UV–vis) spectra were collected using a double‐beam UV–vis spectrophotometer (JASCO V‐750). To ensure data reliability, all electrolyte samples were diluted to a consistent ratio before measurement. The size, thickness, and morphology of the samples were characterized using field‐emission scanning electron microscopy (FE‐SEM; JEOL JMS‐7000F). Crystal structure and crystallographic orientation of electrodeposited samples were analyzed by X‐ray diffraction (XRD, PANalytical Empyrean) with a Cu Kα radiation source (λ = 1.5406 Å). Optical microscopy (Smartzoom 5, Zeiss) was used for in situ observation of the Zn deposition behavior, while three‐dimensional images of the Zn electrodes were acquired using scanning confocal laser microscopy (Keyence VK‐2000). X‐ray photoelectron spectroscopy (XPS) measurements were conducted with a Thermo Scientific Kα instrument, with the C 1s peak of adventitious carbon calibrated to 284.6 eV. Electrochemical analyses were performed using a potentiostat/galvanostat (VMP3, Bio‐Logic).

### 4.5 Electrochemical Measurements

Electrochemical characterization of the Zn||Ti asymmetric cells, Zn||Zn symmetric cells, and Zn||I_2_ full cells were conducted using CR2032‐type coin cells and 3.0 × 4.0 cm^2^ sized pouch‐type cells. The symmetric cells consisted of a 14 mm‐diameter Zn metal electrode (thickness of 50 µm), a Whatman GF/C 1822‐150 glass fiber separator, and 100 µL of 2 M ZnSO_4_ blank electrolyte with TPEN. The asymmetric cells were assembled with a 14 mm‐diameter Ti metal electrode (thickness of 10 µm), a 14 mm‐diameter Zn metal electrode (thickness of 50 µm), a Whatman GF/C 1822‐150 glass fiber separator, and 100 µL of 2 M ZnSO_4_ blank electrolyte and electrolyte with TPEN. In the coin‐type full‐cell batteries, the positive electrode utilized 12 mm‐diameter I_2_@AC dry electrode with I_2_‐loading mass of 29.58 mg cm^−2^, while the negative electrode employed bare Zn (thickness of 30 µm), both with a 2 M ZnSO_4_ blank electrolyte and electrolyte with TPEN with E/C ratio of 12 g Ah^−1^ and N/P ratio of 3.0‐3.4. The galvanostatic charge/discharge performance, rate performance, voltage profiles, and Coulombic efficiency were evaluated using a galvanostatic cycler (TOYO, Japan) under ambient environment and elevated temperature (45°C).

For the single‐layer Zn||I_2_ pouch‐type cell, the assembly sequence from bottom to top is as follows: 20 µm Zn anode, separator, I_2_ cathode. The free‐standing dry electrode was pressed onto a Ti foil (30 × 40 mm), with a loading of I_2_ were approximately 29.58 mg cm^−2^. Glass fiber separator (Whatman, GF/C 1822‐150) and a 2 M ZnSO_4_ aqueous solution with and without TPEN served as the separator and electrolyte. The electrochemical performance of Zn||I_2_ pouch cells were conducted at a potential range of 0.6–1.6 V under the ambient environment with E/C ratio of 6 g Ah^−1^ and N/P ratio of 1.93.

Electrochemical impedance spectroscopy (EIS), linear sweep voltammetry (LSV), chronopotentiometry (CP), chronoamperometry (CA), and cyclic voltammetry (CV) tests were conducted using a Bio‐Logic VMP‐3 electrochemical station. EIS was performed across a frequency range from 1 MHz to 100 mHz with an amplitude of 5 mV using a Bio‐Logic VMP‐3 electrochemical station. CP, CA, and LSV plots were generated employing a three‐electrode system consisting of Zn metal foil as the counter electrode, a saturated calomel electrode as the reference electrode, and Zn metal foil and Ti metal foil as the working electrodes.

### 4.6 In Situ pH Measurements

In‐situ pH tests were conducted in a custom‐made Zn||Zn symmetric cell. The setup included a Zn metal electrode measuring 18 mm in diameter (with a thickness of ∼0.05 mm) and 3 mL of 2 M ZnSO_4_ electrolyte, with and without TPEN. The pH meter (Orion Star A211) and the microelectrodes were procured from Thermo Fisher Scientific.

### 4.7 Theoretical Calculation

Density functional theory (DFT) calculations on isolated molecular species were performed with ORCA 6.0.1. H_2_O, TPEN, TCC, and [Zn(H_2_O)_6_]^2+^ were subjected to geometry optimization at the PBE‐D3(BJ)/def2‐SVP level using the RIJCOSX approximation, the def2/J auxiliary basis set, DEFGRID3, the TightSCF criterion, and CPCM(water). Kohn‐Sham frontier molecular orbital energies were obtained from PBE‐D3(BJ)/ma‐def2‐TZVP single‐point calculations using RIJCOSX, AutoAux‐generated auxiliary basis sets, DEFGRID3, the VeryTightSCF criterion, and CPCM(water). H_2_O and TPEN were treated as neutral closed‐shell singlets, whereas TCC and [Zn(H_2_O)_6_]^2+^ were treated as doubly charged closed‐shell singlets.

Periodic DFT calculations were performed with the Quickstep module of CP2K 2026.1 using the Gaussian and plane waves (GPW) method. The PBE exchange‐correlation functional was combined with the zero‐damping D3 dispersion correction, GTH‐PBE pseudopotentials, and DZVP‐MOLOPT basis sets. Plane‐wave and relative cutoffs of 500 and 60 Ry, respectively, were applied. Zn(002), Zn(100), and Zn(101) slabs, each containing 144 Zn atoms in four atomic layers, were considered. The bottom two Zn layers were fixed, whereas the upper two Zn layers and the adsorbate were allowed to relax. Periodic boundary conditions and the periodic Poisson solver were used, and a surface dipole correction was applied along the z direction. The self‐consistent field (SCF) procedure employed standard diagonalization together with Fermi‐Dirac smearing at 300 K and Broyden density mixing. The SCF convergence threshold was set to 1.0 × 10^−6^ Hartree. Geometry optimizations were performed with the L‐BFGS algorithm using target maximum and root mean square force thresholds of 4.5 × 10^−4^ and 3.0 × 10^−4^ Hartree/Bohr, respectively. The periodic calculations represent idealized vacuum Zn interfaces.

For H_2_O, the adsorption energy was defined as:

(1) $$E_{ads}\;\left(H_{2}O\right)=E_{slab+H_{2}O\;}-\;E_{slab}\;-\;E_{H_{2}O}$$

where all terms were evaluated using CP2K at the same electronic‐structure level. For the dicationic adsorbates A^2+^ (TCC or [Zn(H_2_O)_6_]^2+^), two Cl^−^ counterions were included to construct an overall charge‐neutral periodic cell. The adsorbate was placed above the Zn slab, whereas the two Cl^−^ counterions were placed below the slab and fixed during geometry optimization. The corresponding counterion‐neutralized adsorption energy was defined as:

(2) $$E_{ads}^{2Cl^{-}}=E_{slab+A^{2+}+2Cl^{-}}-\;E_{slab}^{sp}-\;E_{A^{2+}+2Cl^{-}}$$

where $E_{\mathrm{slab}}^{\mathrm{sp}}$ represents the single‐point energy of the Zn slab evaluated in the corresponding simulation cell, and $E_{A^{2+}+2Cl^{-}}$ represents the adsorbate‐counterion reference energy evaluated without the Zn slab. The reference calculation employed nonperiodic boundary conditions and the Martyna‐Tuckerman Poisson solver. By placing the dication and Cl^−^ counterions on opposite sides of the slab, we isolated the dication‐surface interaction while maintaining the neutrality of each simulation. Consequently, the calculated counterion‐neutralized adsorption energy was considered to reflect the dication‐surface interaction, with residual counterion contributions assumed to be negligible.

Polyiodide binding calculations were performed using ORCA 6.0.1 for H_2_O, TPEN, TCC, and [Zn(H_2_O)_6_]^2+^ with I_3_^−^ and I_5_^−^. The isolated hosts, polyiodides, and host‐polyiodide complexes were optimized at the B3LYP‐D3BJ/def2‐SVP level using the RIJCOSX approximation, the def2/J auxiliary basis set, the TightSCF criterion, and CPCM(water). Final electronic energies were obtained from B3LYP‐D3BJ/ma‐def2‐TZVP single‐point calculations using the optimized geometries and the same solvent model. All systems were treated as closed‐shell singlets with their corresponding total charges. The polyiodide binding energy was defined as:

(3) $$E_{bind}=E_{complex}\;-\;E_{host}\;-\;E_{polyiodide}$$

## Author Contributions

**Dong Wook Kim**: conceptualization, methodology, data curation, investigation, validation, formal analysis, visualization, writing – original draft, project administration. **Seung Hwa Park**: methodology, validation, visualization, software, conceptualization, writing – review and editing, writing – original draft. **Tae Rim Kim**: validation, data curation, visualization, conceptualization. **Gun Jang**: visualization, validation, formal analysis. **Jun Su Kim**: validation, formal analysis, visualization. **Myung‐Jun Kwak**: visualization, formal analysis, validation. **Seokhwan Jin**: software. **Seongbin Ga**: software, validation, writing – review and editing. **Chihyun Hwang**: writing – review and editing, validation, investigation, formal analysis, data curation, supervision, project administration. **Ho Seok Park**: writing – review and editing, project administration, supervision, investigation, formal analysis. **Youngkwon Kim**: funding acquisition, writing – review and editing, project administration, resources, supervision, investigation, formal analysis.

## Funding

National Research Foundation of Korea (NRF) grant funded by the Ministry of Science and ICT (MSIT, Korea) (RS‐2024‐00407015, RS‐2024‐00453815 and RS‐2021‐NR061788).

## Conflicts of Interest

The authors declare no conflicts of interest.

## Supporting information




**Supporting File**: advs77565‐sup‐0001‐SuppMat.docx.

## Data Availability

The data that support the findings of this study are available from the corresponding author upon reasonable request.
