# Supplementary material for: Chelation‐Driven Dual‐Interface Regulation for Long‐Life Aqueous Zn–I2 Batteries
Source: Adv Sci (Weinh). 2026 Sep 8:e77565. Online ahead of print. doi: 10.1002/advs.77565 (PMC13554500; doi:10.1002/advs.77565)
Supplement: Supplementary file 1 [file ADVS-9999-e77565-s001.docx]

Supporting Information

**Chelation-Driven Dual-Interface Regulation for Long-Life Aqueous Zn–I_2_ Batteries**

Dong Wook Kim^1^, Seung Hwa Park^1^, Tae Rim Kim^1^, Gun Jang, Jun Su Kim, Myung-Jun Kwak, Seokhwan Jin, Seongbin Ga, Chihyun Hwang^*^, Ho Seok Park^*^, Youngkwon Kim^*^

* Addresses correspondence to chwang@keti.re.kr (C. Hwang); phs0727@skku.edu (H. S. Park); ykkim96@keti.re.kr (Y. Kim).

**Figure S1.** Optimized geometry of Zn^2+^-TPEN chelating complex using DFT calculation.

**Figure S2.** UV-vis spectra of various solutions including TPEN.

**Figure S3.** UV-vis spectra of electrolyte with different concentration of TPEN.

**Figure S4.** Photographs of solubility test of TPEN in electrolyte.

**Figure S5.** Zn||Zn symmetric cell performance using various concentrations of TPEN electrolyte.

**Figure S6.** UV-vis spectra of various solutions after mixing with polyiodide solution.

**Figure S7.** Chronoamperometry measurement using 2M ZS with and without TPEN, respectively.

**Figure S8.** Post-mortem analysis of Raman spectra after cycling using 2 M ZS with and without TPEN, respectively.

**Figure S9.** In-situ pH measurement of Zn||Zn cells using 2 M ZS and 2 M ZS with TPEN electrolytes.

**Figure S10.** pH buffering test of 2 M ZS and TPEN electrolyte.

**Figure S11.** Raman spectra of (a) 2 M ZS, and (b) TPEN electrolyte. (c) O-H band deconvolution results of Raman spectra.

**Figure S12.** Linear sweep voltammograms of Zn||Ti cell.

**Figure S13.** Adsorption energy on Zn slabs of (a) H_2_O, and (b) TCC using DFT calculation.

**Figure S14.** XRD patterns of Zn metal surface after 24 h immersion test.

**Figure S15.** Coulombic efficiency (CE) plots of Zn||Ti asymmetric cell using 2 M ZS and TPEN electrolyte.

**Figure S16.** Rate performance test of Zn||Zn symmetric cells in 2 M ZS and TPEN electrolyte.

**Figure S17.** XRD patterns of Zn metal after plating using 2 M ZS and TPEN electrolyte, respectively.

**Figure S18.** XRD pattern after Zn deposition using (a) 2 M ZS, and (b) TPEN electrolyte. (c) RTC calculation results.

**Figure S19.** SEM images of Zn deposits in 2 M ZS with and without TPEN electrolyte after 8 mAh cm^−2^ deposition.

**Figure S20.** Photographs for the mixture of 2 M ZS electrolyte and polyiodide solution.

**Figure S21.** Raman spectra of solid precipitate powder.

**Figure S22.** Raman spectra of various mixtures based on NaI_x_ solution.

**Figure S23.** Binding energy of various species with polyiodide using DFT calculation.

**Figure S24.** UV-vis spectra of various solutions after mixing with polyiodide solution.

**Figure S25.** Raman spectra of TPEN electrolyte during charging.

**Figure S26.** UV-vis spectra of TPEN electrolyte after cycling.

**Figure S27.** Cyclic voltammogram at various scan rates using a) 2 M ZS, and b) TPEN electrolyte. c) Relationship between peak current density and square root of the scan rate for Zn||I2 batteries in 2 M ZS with and without TPEN electrolyte.

**Figure S28.** Quasi-OCV measurement of Zn||I_2_ batteries using a) 2 M ZS blank electrolyte and b) 2 M ZS with TPEN electrolyte.

**Figure S29.** Post-mortem analysis of Raman spectra of I_2_ positive electrode after 100 cycles of high-loading Zn||I_2_ batteries using 2 M ZS and TPEN electrolyte.

**Figure S30.** Zn||I_2_ cell performance under high-temperature condition.

**Figure S31.** (a) Photograph of the 3 cm × 4 cm Zn||I_2_ pouch cell. (b) Zn||I_2_ pouch cell cycling performance using 2 M ZS and TPEN electrolyte. (c) Cycling profiles of three independently assembled pouch cells using TPEN electrolyte tested at 1 mA cm^-2^ (≈ 0.17 C), an N/P ratio of 1.93, and an E/C ratio of 6 g Ah^-1^.

**Figure S32.** Schematic of the comparison of operating mechanism of the TCC additive in Zn–I_2_ batteries.

**Table S1** The comparison of electrochemical performance of Zn–I_2_ batteries with reported works.

| **Name** | **I_2_ loading level**  **[mg cm^-2^]** | **C-rate** | **Capacity retention** | **Ref.** |
| --- | --- | --- | --- | --- |
| TPEN | 29.58  29.58 | 2  0.18 | 88.35% @ 700 cycles  92.5% @ 200 cycles | This work |
| RSC[1] | 12.7 | 3 | 97.95% @ 200 cycles | 1 |
| VB6[2] | 7 | 1.8 | 76.5% @ 450 cycles | 2 |
| TMAI[3] | 15 | 0.33 | 91.3% @ 100 cycles | 3 |
| HMTA[4] | 18.5 | 2.1 | 80% @ 672 cycles | 4 |
| AEC[5] | 15.7 | 5.6 | 86.68% @ 682 cycles | 5 |
| BMT[6] | 15.5 | 11.8 | 76.2% @ 800 cycles | 6 |
| DESA-E[7] | 16.67 | 1 | 88.15% @ 200 cycles | 7 |
| ZCIM[8] | 14.7 | 2.5 | 89% @ 200 cycles | 8 |
| BmBr[9] | 11.85 | 0.25 | 95.8% @ 100 cycles | 9 |
| AZPY[10] | 22.8 | 2.2 | 96.3% @ 150 cycles | 10 |

**Table S2** The comparison of electrochemical performance of Zn–I_2_ pouch-type batteries with reported works.

| **Name** | **Additive**  **concentration** | **Areal capacity**  **[mAh cm-2]** | **Zn thickness**  **(μm)** | **N/P ratio** | **Current density** | **Ref.** |
| --- | --- | --- | --- | --- | --- | --- |
| TPEN | 0.01 wt% | 6 | 20 μm | 1.93 | 1.7 mA cm^-2^ | **This work** |
| DPL[11] | 0.1 wt% | 2.5 | 17 μm | 3.8 | 0.5 A g^-1^ | 1 |
| HMTA[4] | 0.2 M | 2.8 | 10 μm | 2.14 | 0.4 A g^-1^ | 2 |
| ChCl[12] | 0.05 M | 3.63 | 20 μm | 3.3 | 2 A g^-1^ | 3 |
| Ect[13] | 5 wt% | 3.47 | 20 μm | 1.25 | 1.7 mA cm^-2^ | 4 |
| AMI[14] | 10 mM | 3.1 | 7.5 μm | 1.88 | 2 A g^-1^ | 5 |
| PUB[15] | 0.6 M | 1.65 | 8.5 μm | 3.0 | 2 A g^-1^ | 6 |

**References**

[1]	Z. Chen, X. Gao, L. Shan, Q. Fu, Z. Xing, P. Rao, Z. Kang, X. Shi, W. Zhang, X. Tian, *Energy & Environmental Science* **2025**, *18* (19), 8768.

[2]	X. Yang, L. Zhang, J. Zhu, L. Wang, Y. Zhang, Z. Zhai, J. Kang, Y. Shao, J. Zhang, X. Zhang, *Energy Storage Materials* **2025**, *78*, 104268.

[3]	J. Kang, J. Zhang, W. Wan, Z. Zhai, G. Liu, Y. Ge, L. Wang, C. Wang, H. Lu, *Energy & Environmental Science* **2025**, *18* (10), 4800.

[4]	S.-J. Zhang, J. Hao, H. Wu, Q. Chen, Y. Hu, X. Zhao, S.-Z. Qiao, *Journal of the American Chemical Society* **2025**, *147* (19), 16350.

[5]	W. Li, M. Chen, Q. Zhou, X. Mi, H. Zhao, B. Liu, J. Chen, Y. Yao, *Advanced Functional Materials* **2026**, *36* (4), e13773.

[6]	B. Qi, Y. Chai, Y. Hu, Z. Liu, Y. Wang, K. Chen, C. Tan, X. Bai, K. Xie, H. Cheng, *Angewandte Chemie International Edition* **2025**, *64* (33), e202507497.

[7]	X. Chen, D. Feng, Y. Jiao, P. Wu, *Advanced Materials* **2026**, *38* (6), e13389.

[8]	B. Ren, Y. Pan, Y. Xie, Y. Jiao, P. Wu, *ACS Materials Letters* **2025**, *7* (10), 3394.

[9]	Y. Lv, C. Huang, M. Zhao, M. Fang, Q. Dong, W. Tang, J. Yang, X. Zhu, X. Qiao, H. Zheng, *Journal of the American Chemical Society* **2025**, *147* (10), 8523.

[10]	P. Yang, J. Guo, S. Lin, Y. Fu, W. Guo, *Journal of the American Chemical Society* **2025**, *147* (34), 30820.

[11]	X. Fu, Y. Pan, Z. Chen, F. Li, Y. Yang, M. Chen, H. Tu, T. Qiu, Z. Xing, P. Rao, *Energy & Environmental Science* **2026**, *19* (4), 1170.

[12]	X. Bi, Y. Ren, A. Yu, L. Yang, R. He, Y. Cheng, X. Chang, X. Lu, Y. Xie, Q. Xue, *Advanced Functional Materials* **2026**, e76224.

[13]	Y. Hu, S.-J. Zhang, H. Wu, Q. Chen, P. Zhang, J. Hao, S.-Z. Qiao, *Energy & Environmental Science* **2026**, *19* (5), 1551.

[14]	Y. Tan, W. Xu, F. Yang, J. Tao, D. Li, Y. Zhang, F. Huang, B. Tan, H. He, *Advanced Materials* **2026**, *38* (12), e22609.

[15]	G. Ou, B. Li, P. Liu, M. Ye, Z. Wen, Y. Zhang, Y. Tang, X. Liu, C. C. Li, *Advanced Energy Materials* **2026**, e71015.
